# The LC-QTOF-MS/MS analysis of acid degradation products of Rifaximin, an antibiotic

**DOI:** 10.1016/j.mex.2022.101735

**Published:** 2022-05-23

**Authors:** Saima Baig Yaseen, Arfa Akram, Syed Ghulam Musharraf, Mehwish Wajidi, Nargis Tabassum, Nemat Nazir, Syed Muhammad Zaki Shah

**Affiliations:** a Federal Urdu University of Arts Sciences and Technology, Karachi, Sindh Pakistan; b H.E.J Research Institute of Chemistry, International Center for chemical and Biological Sciences, University of Karachi, Karachi 75270, Pakistan; c Dr. Panjwani Center for Molecular Medicine and Drug Research, International Center for Chemical and Biological Sciences, University of Karachi, Karachi 75270, Pakistan

**Keywords:** Analysis LCMS/MS-QTOF, Stress degradation, Bruker

## Abstract

The present research aims to propose a simple and accurate technique for the analysis of Rifaximin in the presence of its stress degradation products and analysis of degradation products by LC-MS/MS analysis. Rifaximin was submitted to forced degradation under the acid hydrolysis condition as prescribed by the ICH. The extract was prepared by firstly treated with HCl and heated about 4 to 8 h. The filtrate was collected and separated using dichloromethane followed by evaporation in rotary evaporator to obtain a solid crude extract which was then stored under refrigeration at -80 °C. Liquid chromatography quadrupole time-of-flight mass spectrometry (LC-QTOF-MS/MS) was utilized to identify products in the drug sample. The data processing results revealed the presence of 9 products in the degraded sample of Rifaximin. This data article contains the *m/z* [*M* + *H* +] values, molecular formula, retention times and the comprehensive list of *m/z* values detected during the LC- QTOF- MS/MS analysis.

## Graphical abstract

**Figure:**
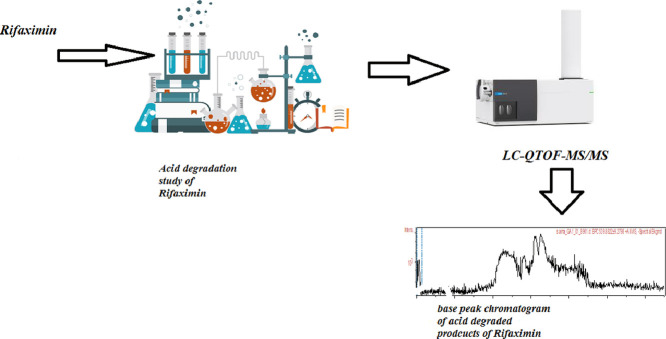

Specifications table

**Table:** 

Subject Area	Pharmacy
More specific subject area	Pharmaceutical Chemistry
Type of data How Data was acquired	Tables and Figures Data was acquired using liquid chromatography mass spectrometry (LCMS) through a Phenomenex Jupitor 5μm C18 300Å 5.0×100mm coupled to a CompactTM Q-TOF(Bruker daltonics,Bremen, Germany)
Data format Experimental factors	Analyzed data Products of drug was forced degraded with dichloromethane and concentrated at 65 °C
Experimental features	Identified products proﬁling in acid degradation was performed
Method Name	LC- QTOF-MS/MS analysis
Name and reference of original method	Data of the products obtained using LC- QTOF-MS/MS analysis and the comprehensive list of detected products is available in Table 3
Resource availability	The active rifaximin was complementary provided by Hilton Pharma Pvt Ltd; acid degradation of rifaximin, LC-QTOF-MS/MS analysis and data processing was done at the H.E.J research Institute of Chemistry, University of Karachi
Related research article	Mahadik, M., Bhusari, V.,Kulkarni, M., & Dhaneshwar, S.LCUV and LCMS evaluation of stress degradation behavior of tenatoprazole. Journal of pharmaceutical and biomedical analysis. 50(2009),787-793 [1]

## Method details

### Collection and preparation of acid degraded products

About 1 gm of Rifaximin drug was collected from Hilton Pharma Pvt Ltd. The drug sample was immediately transferred to the laboratory after collection and stored in refrigerator. About 200 mg of sample was taken and dissolve in 200 mL of methanol. Added 200 mL of 0.1 M HCl in it and transferred it in a round bottom flask using a parallel synthesizer i-e, using condenser fitted with chiller and heating mental. The solution was refluxed for about 4 to 8 h at 60 °C. Then after 4–8 h cool down at room temperature and transferred the solution in a separating funnel. Added 50 mL of dichloromethane, shake it well until the layer separated then collected lower layer. The process was repeated by 3 times to collect the layer. Then the filtrate was transferred into a round bottom flask and evaporate it on a rotary evaporator, and collected the dry product after evaporation. The resulting solid crude was then kept in frozen storage at -80 °C.

**Base Peak Chromatogram ofRifaximinstandard solution.**

### Data

**Fig. 1:**
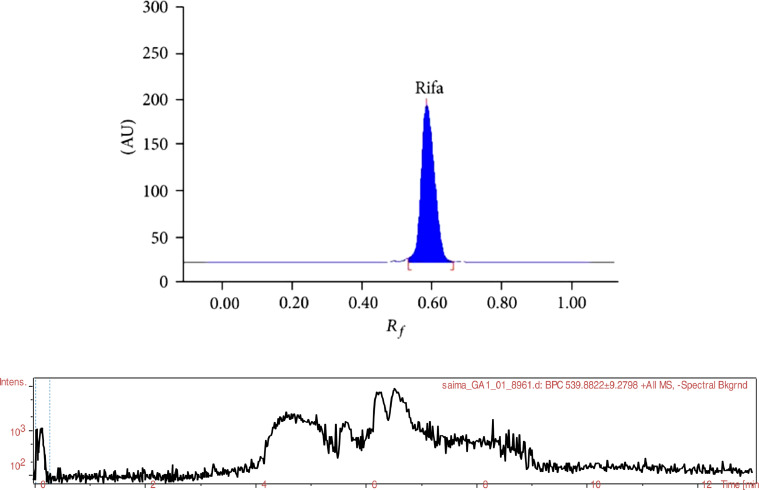
Base peak chromatogram (BPC) of the acid degraded rifaximin solution.

Figure 1 shows the base peak chromatogram of Rifaximin solution which was obtained by analyzing acid degraded sample of rifaximin using LC-QTOF-MS/MS. The data of 9 proposed products which includes the measured *m/z* [*M* + *H*^+^] values, calculated *m/z*, retention time (RT min) detected during the LC-QTOF-MS/MS analysis are available in the table A.

### Degraded products profiling by LC-QTOF-MS/MS

Products profiling of the drug solution was done by using LC-QTOF- MS/MS in positive mode (ESI+). The analysis was done firstly by dissolving 1 mg of the drug powder in 1 mL of analytical grade methanol followed by sonicating for 05 min, and finally centrifuge for 10 min at 12,000 RPM. A sample injection volume of 5 mL was used for chromatographic separation of analytes in reverse phase ultra-high-performance liquid chromatography (RP- UHPLC) through a Zorbax Eclipse plus C-18 column 1.8 µm with dimensions of 2.1 mm (internal diameter), 50 mm (length). The analytical run was set at 20 mins. The flow profile of the mobile phase is shown in Table 1. Other parameters of the LC- QTOF-MS/MS system [2], [3] are summarized in Table 2.

**Table 1:** Isocratic and gradient flow profiles of the mobile phase.

Time (minutes)	Flow (µL/minute)	Solvent%
0–1	300	10
1–5	300	70
5–9	300	90
9–20	300	10

**Table 2:** Parameters of the LC- QTOF-MS/MS system.

Acquisition	Parameter
Source type	Electrospray-ionization
Ion polarity	Positive
Scan	50–1500 *m/z*
Set capillary	4500 V
Set end plate offset	+500V
Set nebulizer	2.8 bar
Set dry heater	300 °C
Set dry gas	10/minute

**Table 3:** Data analysis for products profiling was done using Bruker Compass Data Analysis software version 4.4 (Bruker Daltonics, Bremen, Germany).

S.No	Experimental *m/z*	Measured mass	RT(min)	Ion Formula	Bruker data analysis peak
**01-**	767.12	**767.01**	**0.1**	**C_42_H_4_**_4_**N_3_O_11_**	
**02-**	**724.23**	**724.31**	**0.1**	**C_40_H_4_**_1_**N_3_O_10_**	
**03-**	**643.32**	**643.52**	**0.1**	**C_36_H_4_**_0_**N_3_O_8_**	
**04-**	**585.14**	**585.44**	**0.1**	**C_34_H_3_**_8_**N_3_O_6_**	
**05-**	567.13	**567.12**	**0.1**	**C_34_H_3_**_6_**N_3_O_5_**	
**06-**	**560.11**	**560.20**	**0.2**	**C_34_H**_29_**N_3_O_5_**	
**07-**	**485.11**	**485.12**	**6.3**	**C_30_H_1_**_6_**N_2_O_5_**	
**08-**	**349.13**	**349.30**	**6**	**C_24_H_1_**_6_**N_2_O**	
**09-**	**180.10**	**180.01**	**5.6**	**C_13_H**_9_**N**	

## Ethics statements

No any ethical statement.

## CRediT author statement

Dr Syed Ghulam Musharraf**:** Conceptualization, Methodology; Saima Yaseen Baig**:** Software, Data curation, Writing- Original draft preparation; Saima Yaseen Baig, Dr Arfa Akram, Dr Mehwish Wajidi**:** Visualization, Investigation; Dr Ghulam Musharraf, Dr Arfa Akram**:** Supervision; Saima Yaseen Baig, Dr Nargis tabassum**:** Software, Validation; Syed Muhammad Zaki Shah, Nemat Nazir**:** Writing- Reviewing and Editing.

## Declaration of Competing Interests

The authors declare that they have no known competing financial interests or personal relationships that could have appeared to influence the work reported in this paper.
